# Outcomes in minor stroke patients treated with intravenous thrombolysis

**DOI:** 10.1111/cns.14164

**Published:** 2023-03-21

**Authors:** Chunmiao Duan, Yunyun Xiong, Hong‐Qiu Gu, Shang Wang, Kai‐Xuan Yang, Manjun Hao, Xingquan Zhao, Xia Meng, Yongjun Wang

**Affiliations:** ^1^ Department of Neurology, Beijing Tiantan Hospital Capital Medical University Beijing China; ^2^ China National Clinical Research Center for Neurological Diseases Beijing China; ^3^ Department of Neurology, Beijing Daxing Hospital Capital Medical University Beijing China; ^4^ Chinese Institute for Brain Research Beijing China; ^5^ National Center for Healthcare Quality Management in Neurological Diseases Beijing China; ^6^ Neurocardiology Center, Department of Neurology, Beijing Tiantan Hospital Capital Medical University Beijing China; ^7^ Center for Stroke Beijing Institute for Brain Disorders Beijing China

**Keywords:** minor stroke, outcome, stroke, thrombolysis

## Abstract

**Aims:**

Our study aimed to describe the short‐, medium‐, and long‐term outcomes of intravenous thrombolysis in minor stroke, and to explore the relationship between thrombolysis and clinical outcomes.

**Methods:**

Our study included ischemic minor stroke patients (National Institutes of Health Stroke Scale score ≤ 5) within 4.5 h from symptom onset from the Third China National Stroke Registry (CNSR‐III) between August 2015 and March 2018. The primary outcome was a favorable functional outcome, defined as a modified Rankin Scale (mRS) score of 0–1 at 3 months. The secondary outcomes included mRS score of 0–1 at discharge, 6 months, and 1 year. The safety outcomes were symptomatic intracerebral hemorrhage (sICH) at 24–36 h and all‐cause mortality. The association between intravenous thrombolysis and clinical outcomes was studied using multivariable models.

**Results:**

A total of 1905 minor ischemic stroke patients were included. Overall 527 patients (28%) received intravenous t‐PA (IV t‐PA) and 1378 patients (72%) in the non‐IV t‐PA group. Of them, 18.85% (359/1905) participants had a disabled outcome (defined as mRS score ≥ 2) at discharge, 12.8% (242/1885) at 3 months, 13.9% (262/1886) at 6 months, and 13.9% (260/1871) at 1 year. In multivariable analysis, IV t‐PA was associated with favorable functional outcomes at discharge (adjusted odds ratio [aOR] 1.49; 95% confidence interval [CI] 1.13–1.96; *p* = 0.004), 3 months (aOR 1.51; 95% CI 1.09–2.10; *p* = 0.01), 6 months (aOR 1.64; 95% CI 1.19–2.27; *p* = 0.003), and 1 year (aOR 1.52; 95% CI 1.10–2.10; *p* = 0.01). Symptomatic ICH occurred in 3 (0.6%) patients in IV t‐PA versus 2 (0.1%) in the non‐IV t‐PA group. No significant differences were found in all‐cause mortality between the two groups.

**Conclusions:**

Intravenous t‐PA may be safe and effective in minor stroke (NIHSS ≤ 5) within a 4.5‐h window and further randomized controlled trials are warranted.

## Graphical abstract

A total of 1905 ischemic minor stroke patients (National Institutes of Health Stroke Scale score ≤ 5) within 4.5 h from symptom onset from the Third China National Stroke Registry (CNSR‐III) between August 2015 and March 2018 were included. Overall 527 patients (28%) received intravenous t‐PA (IV t‐PA) and 1378 patients (72%) in the non‐IV t‐PA group.

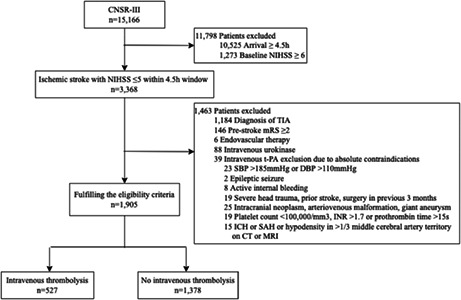

## 1 INTRODUCTION

More than 50% of strokes present with minor stroke (NIHSS ≤ 5) on admission.^1^, ^2^ And about 30% of these patients were unable to ambulate independently at discharge and had a disabled outcome at 90 days.^3^, ^4^ Intravenous alteplase was recommended for minor disabling stroke within 4.5 h window according to the latest guidelines.^5^ Nevertheless, the subjective nature of minor disabling stroke and potential hemorrhagic risk contributed to the discrepancy in clinical practice.^6^, ^7^ Less than half of the patients with minor stroke received intravenous thrombolysis treatment from American cohorts.^3^, ^4^ And the rate of thrombolysis in these populations was even lower in developing and undeveloped countries, including China.^8^

To date, there has been limited evidence of thrombolysis for acute minor ischemic stroke. A meta‐analysis of 6756 patients from contemporary completed randomized phase 3 trials of thrombolysis showed the benefit of intravenous alteplase for minor stroke patients of an NIHSS score of 0–4.^9^ In addition, previous registries reported intravenous thrombolysis was associated with short‐term outcomes including discharge to home, ambulation independently or modified Rankin score (mRS) at discharge.^3^, ^8^, ^10^ Other observational studies found intravenous thrombolysis was associated with 90‐day favorable outcomes in the analysis of relative higher baseline NIHSS.^2^, ^4^, ^11^ However, the Potential of rt‐PA for Ischemic Strokes With Mild Symptoms (PRISMS) trial aimed to evaluate the efficacy and safety of alteplase with non‐disabling minor ischemic stroke (NIHSS ≤ 5) and revealed that alteplase was not superior to aspirin for a 90‐day favorable functional outcome. Instead, alteplase increased the three‐fold risk of symptomatic intracerebral hemorrhage (sICH).^7^ Nevertheless, this trial was terminated early and did not provide strong evidence. So, whether thrombolysis is necessary for all minor stroke is inconsistent. Moreover, data on the association between thrombolysis and medium‐ and long‐term functional outcomes in Asian patients with minor stroke are lacking.

Our study aimed to analyze the short‐, medium‐, and long‐term outcomes of minor stroke with intravenous thrombolysis utilizing a large‐scale prospective registry in China, and identify the association between intravenous alteplase and clinical outcomes.

## 2 METHODS

The CNSR‐III was a nationwide prospective registry that included 15,166 patients with acute ischemic stroke (AIS) and transient ischemic attack (TIA) from 201 hospitals of 22 provinces and four municipalities in China between August 2015 and March 2018, primarily for clarifying the pathogenesis and prognostic factors of ischemic stroke. All patients fulfilling the criteria of age older than 18 years and diagnosis of AIS or TIA within 7 days simultaneously were consecutively enrolled. The study was approved by institutional ethics committees and written informed consent was obtained. The detailed design and main description of the CNSR‐III have been published previously.^12^

### 2.1 Study population

We included the eligible patients fulfilling the following inclusion criteria: (1) minor ischemic stroke, which was defined by NIHSS ≤ 5; (2) time window: defined by presenting with stroke symptoms within 4.5 h of symptom onset or within 4.5 h of awakening after the point when last seen well. The exclusion criteria were (1) admission diagnosis of TIA or with complete symptom relief on initial evaluation; (2) baseline disability: demonstrated by mRS ≥ 2; (3) endovascular therapy including arterial thrombolysis and mechanical thrombectomy; (4) treated with intravenous urokinase; (5) intravenous tissue‐type plasminogen activator (IV t‐PA) exclusion in the whole cohort due to absolute contraindications including despite antihypertensive treatment, persistent blood pressure elevation (systolic > 185 mmHg or diastolic > 110 mmHg); epileptic seizure, hemiplegia after seizures (Todd's palsy); severe head trauma; active internal bleeding or prior stroke or intracranial or intraspinal surgery in previous 3 months; known intracranial neoplasm, arteriovenous malformation, or giant aneurysm; platelet count <100,000/mm^3^; if use of warfarin, INR > 1.7 or prothrombin time >15 s; hypodensity in >1/3 middle cerebral artery territory or intracerebral hemorrhage (ICH) or subarachnoid hemorrhage (SAH) identified by CT or MRI.

### 2.2 Data collection and definitions

Patients were divided into IV t‐PA and non‐IV t‐PA groups based on whether intravenous t‐PA (0.9 mg/kg to a maximum dose of 90 mg, with 10% as an initial bolus and the remaining over one‐hour intravenous infusion) administered within the 4.5‐h time window. We abstracted the following variables: demographics (including age, sex, ethnicity, and insurance), medical history (including current smoking, hypertension, diabetes mellitus, dyslipidemia, coronary heart disease[CHD], atrial fibrillation [AF], prior stroke/TIA, carotid stenosis [defined as the stenosis of extracranial carotid artery ≥50%], peripheral vascular disease [PVD], heart failure), medication history (including antiplatelet, anticoagulant, antihypertensive, glucose‐lowering agents and lipid‐lowering agents), arrival and care (onset to door time, pre‐hospital transportation, care in a stroke unit, fasting blood glucose at admission, systolic blood pressure [SBP] at admission, diastolic blood pressure [DBP] at admission), stroke severity (measured by NIHSS, range of 0–42, with higher scores indicating severe deficit, including its components), etc. The etiology classification of ischemic stroke was defined based on an expanded version of the TOAST (Trial of Org 10172 in Acute Stroke Treatment) classification.^13^ We obtained imaging variables of symptomatic extra‐ and intracranial arterial stenosis (sEICAS), which was defined by 50%–99% stenosis or occlusion of any extra‐ and intracranial artery accounting for the acute infarction lesion and neurological symptoms. The detailed definitions of sEICAS in CNSR‐III were described previously.^14^

### 2.3 Outcomes

The primary outcome of this study was mRS score of 0–1 at 3 months. The secondary outcomes included mRS score of 0–1 at discharge, 6 months, and 1 year. Outcomes were obtained through face‐to‐face interviews at discharge and 3 months and telephone by well‐trained research coordinators at 6 months and 1 year. Regarding the safety outcome, we recorded all‐cause mortality, intracerebral hemorrhage (ICH) which was detected on brain imaging at 24–36 h, and symptomatic intracerebral hemorrhage (sICH) defined as clinical deterioration with an increase in the NIHSS of at least four points or death attributed to hemorrhage on brain imaging, according to the European Cooperative Acute Stroke Study III (ECASS III) criteria.^15^

### 2.4 Statistical analysis

The data were tested for normal distribution using the Kolmogorov–Smirnov test. Continuous variables with normal distribution were described as mean ± standard deviation (SD) or as median with interquartile range (IQR) otherwise, and differences were assessed using the *t*‐test if normally distributed or Mann–Whitney *U* test. Categorical variables were expressed as frequencies with percentages, and the *χ* ^2^ analysis or Fisher exact test was performed to compare the difference between groups. Three different multivariable‐adjusted logistic regression models were used to balance the influence of covariates on the associations between IV t‐PA and dichotomous outcomes. Model 1 adjusted age, sex, ethnicity, and baseline NIHSS. Model 2 additionally adjusted medical insurance, onset to door time, arrival modality, current smoking, hypertension, diabetes mellitus, hyperlipidemia, prior CHD, AF, heart failure, prior stroke/TIA, carotid stenosis, PVD, SBP at admission, stroke unit, antiplatelet, anticoagulant, antihypertensive, lipid‐lowering agents, glucose‐lowering agents, and TOAST subtype. Model 3 adjusted factors in model 2 plus sEICAS ≥ 50%. Subgroup analyses were conducted in patients with a history of baseline NIHSS (0–2 and 3–5), final diagnosis of ischemic stroke, and sEICAS to determine the association of intravenous alteplase with functional outcomes. Last, propensity score matching (PSM) analysis was undertaken to balance the baseline characteristics in the whole cohort. And 1:1 matching was conducted based on the nearest‐neighbor matching technique with a caliper width of 0.25. Two‐sided *p* < 0.05 was indicated as statistically significant. All analyses were carried out using the SAS, version 9.4, software (SAS Institute).

## 3 RESULTS

### 3.1 Patient flowchart

**FIGURE 1:**
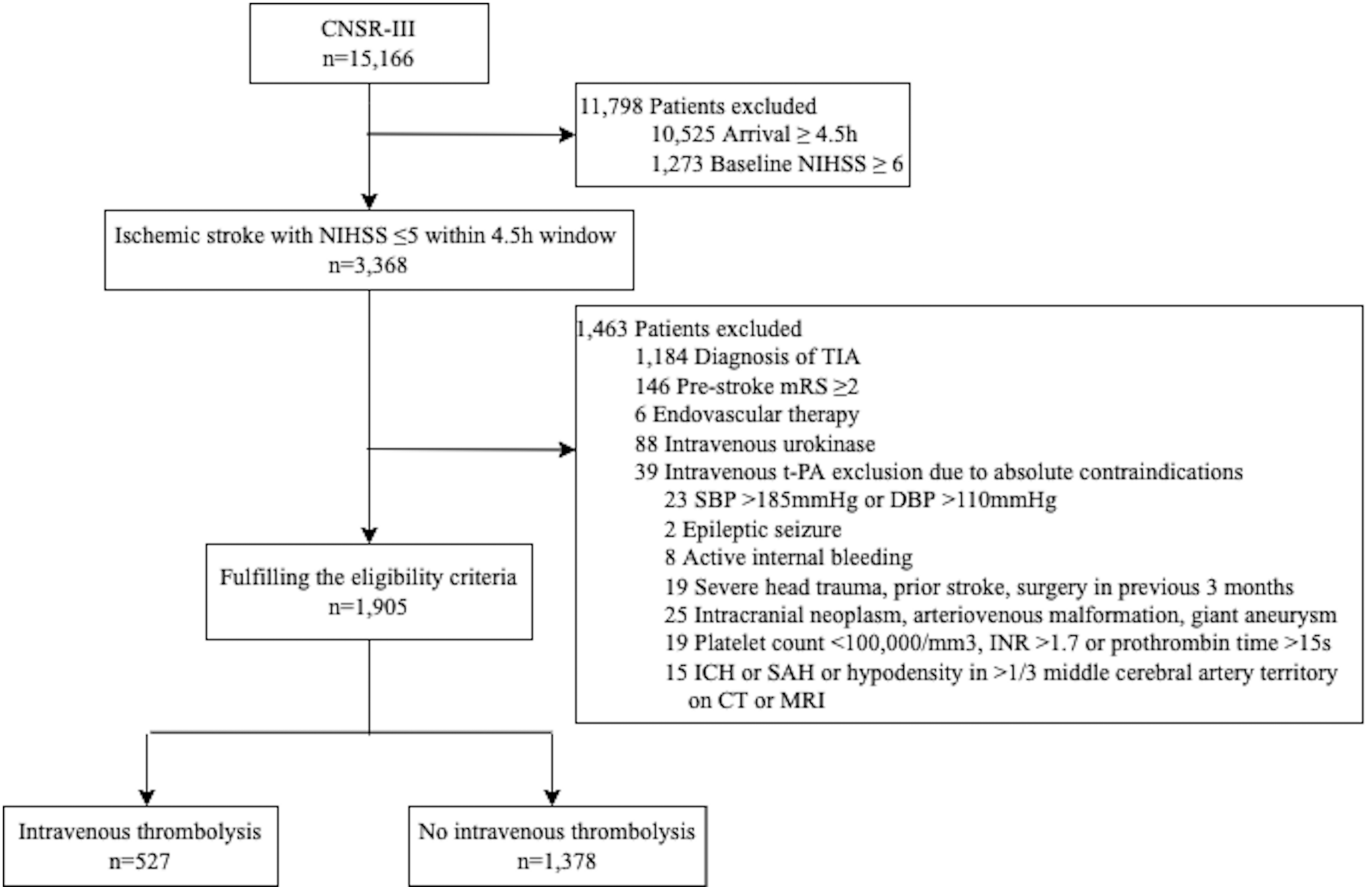
Flowchart. CNSR‐III, the Third China National Stroke Registry; CT, computed tomography; DBP, diastolic blood pressure; ICH, intracerebral hemorrhage; INR, international normalized ratio; MRI, magnetic resonance imaging; mRS, modified Rankin Scale; NIHSS, National Institutes of Health Stroke Scale; SAH, subarachnoid hemorrhage; SBP, systolic blood pressure; TIA, transient ischemic attack; t‐PA, tissue‐type plasminogen activator.

A total of 1905 patients with minor stroke (defined by NIHSS ≤ 5) within a 4.5‐h time window were enrolled for subsequent analysis and the flowchart depicted the detailed reasons for exclusion (Figure 1), including TIA (*n* = 1184), pre‐stroke mRS ≥ 2 (*n* = 146), endovascular therapy including arterial thrombolysis and mechanical thrombectomy (*n* = 6), intravenous urokinase (*n* = 88), and no IV t‐PA due to absolute contraindications (*n* = 39). Finally, 1378 patients (72%) in the non‐IV t‐PA group and 527 patients (28%) received IV t‐PA, in which the final diagnosis was ischemic stroke in 525 patients (99.6%) and TIA in 2 patients (0.4%).

### 3.2 Baseline characteristics

The baseline characteristics of the 1905 patients were described in Table 1. Overall, 76.1% arrived within 3 h from symptom onset, and 23.9% in the 3‐ to 4.5‐h window. The median of onset‐to‐door time was 2.0 h (interquartile range [IQR], 1.1–3.0). The median onset‐to‐door time in the IV t‐PA group was around 18 min earlier than that in the non‐IV t‐PA group. The stroke etiology in those patients whose final diagnosis was ischemic stroke included 21.5% (409/1905) large artery atherosclerosis (LAA), 8.3% (158/1905) cardioembolic, 22.8% (435/1905) small artery occlusion (SAO), 0.9% (17/1905) other determined cause, and 46.5% (886/1905) undetermined cause. The distribution of sEICAS was similar for both groups (23.5% in the IV t‐PA group versus 22.2% in the non‐IV t‐PA group, *p* = 0.34). Patients treated with IV t‐PA were more frequently arrived by emergency medical services (EMS), had higher systolic blood pressure, and had less prior stroke/TIA history. They were also less proportion of taking antiplatelet and lipid‐lowering therapy and a higher proportion of being treated in the stroke unit.

**TABLE 1:** Baseline characteristics of patients based on intravenous alteplase.

Variables	Total (*n* = 1905)	Non‐IV t‐PA (*n* = 1378)	IV t‐PA (*n* = 527)	*p* Value
Age, y	63.0 (55.0–70.0)	63.0 (55.0–70.0)	62.0 (55.0–69.0)	0.71
Female	573 (30.1)	407 (29.5)	166 (31.5)	0.40
Ethnicity				
Han	1856 (97.4)	1340 (97.2)	516 (97.9)	0.41
Non‐Han	49 (2.6)	38 (2.8)	11 (2.1)	
Medical insurance				
Urban worker/public health medical insurance	916 (48.1)	643 (46.7)	273 (51.8)	0.04
Urban resident basic medical insurance	346 (18.2)	260 (18.9)	86 (16.3)	0.20
Rural cooperation medical insurance	523 (27.5)	396 (28.7)	127 (24.1)	0.04
Commercial insurance	9 (0.5)	7 (0.5)	2 (0.4)	0.71
Self‐payment	124 (6.5)	80 (5.8)	44 (8.3)	0.04
Time measure				
Onset to door, hours	2.0 (1.1–3.0)	2.1 (1.2–3.2)	1.8 (1.0–2.5)	<0.001
≤3 h	1449 (76.1)	996 (72.3)	453 (86.0)	
3–4.5 h	456 (23.9)	382 (27.7)	74 (14.0)	
Arrival modality				
Arrival by EMS	294 (15.4)	171 (12.4)	123 (23.3)	<0.001
Arrival self	1190 (62.5)	896 (65.0)	294 (55.8)	
Arrival others	421 (22.1)	311 (22.6)	110 (20.9)	
Medical history				
Current smoking	600 (31.5)	428 (31.1)	172 (32.6)	0.51
Hypertension	1183 (62.1)	841 (61.0)	342 (64.9)	0.12
Diabetes mellitus	432 (22.7)	322 (23.4)	110 (20.9)	0.24
Dyslipidemia	155 (8.1)	116 (8.4)	39 (7.4)	0.47
Prior CHD	222 (11.7)	168 (12.2)	54 (10.2)	0.24
AF	154 (8.1)	114 (8.3)	40 (7.6)	0.62
Heart failure	14 (0.7)	11 (0.8)	3 (0.6)	0.60
Prior Stroke/TIA	400 (21.0)	310 (22.5)	90 (17.1)	0.01
Carotid stenosis	22 (1.2)	15 (1.1)	7 (1.3)	0.66
PVD	20 (1.0)	17 (1.2)	3 (0.6)	0.20
Fasting glucose at admission, mmol/L	5.5 (4.9–6.8)	5.5 (4.9–6.8)	5.5 (4.8–6.8)	0.34
SBP at admission, mmHg	150.0 (136.5–165.0)	149.5 (135.0–164.0)	150.0 (140.0–166.0)	0.02
DBP at admission, mmHg	85.5 (79.5–95.0)	85.0 (79.0–95.0)	87.5 (79.5–95.0)	0.21
Baseline NIHSS	2.0 (1.0–4.0)	2.0 (1.0–3.0)	3.0 (2.0–4.0)	<0.001
0	281 (14.8)	244 (17.7)	37 (7.0)	
1	417 (21.9)	327 (23.7)	90 (17.1)	
2	375 (19.7)	267 (19.4)	108 (20.5)	
3	310 (16.3)	215 (15.6)	95 (18.0)	
4	309 (16.2)	199 (14.4)	110 (20.9)	
5	213 (11.2)	126 (9.1)	87 (16.5)	
Care in stroke unit	534 (28.0)	313 (22.7)	221 (41.9)	<0.001
TOAST subtype				
LAA	409 (21.5)	295 (21.4)	114 (21.6)	0.94
Cardioembolic	158 (8.3)	113 (8.2)	45 (8.5)	
SAO	435 (22.8)	319 (23.1)	116 (22.0)	
Other determined cause	17 (0.9)	11 (0.8)	6 (1.1)	
Undetermined cause	886 (46.5)	640 (46.4)	246 (46.7)	
Imaging marker				
sEICAS ≥50%	430 (22.6)	306 (22.2)	124 (23.5)	0.34
Medication history				
Antiplatelet	342 (18.0)	265 (19.2)	77 (14.6)	0.02
Anticoagulant	16 (0.8)	13 (0.9)	3 (0.6)	0.42
Antihypertensive	864 (45.4)	615 (44.6)	249 (47.2)	0.30
Lipid‐lowering agents	223 (11.7)	173 (12.6)	50 (9.5)	0.06
Glucose‐lowering agents	345 (18.1)	258 (18.7)	87 (16.5)	0.26

Abbreviations: AF, atrial fibrillation; CHD, coronary heart disease; DBP, diastolic blood pressure; EMS, emergency medical services; IV t‐PA, intravenous tissue‐type plasminogen activator; LAA, large artery atherosclerosis; NIHSS, National Institutes of Health Stroke Scale; PVD, peripheral vascular disease; SAO, small artery occlusion; SBP, systolic blood pressure; sEICAS, symptomatic extra‐intracranial atherosclerotic stenosis; TIA, transient ischemic attack; TOAST, Trial of ORG 10172 in Acute Stroke Treatment.

Those treated with IV t‐PA had higher baseline NIHSS (mean ± SD, 2.8 ± 1.5 versus 2.1 ± 1.6). A higher NIHSS score of 3–5 was recorded in 55.4% of those treated with IV t‐PA than in 39.1% of those not treated. The top five neurological deficits defined by the baseline NIHSS subitems, were facial palsy (40.68%), lower limb weakness (36.58%), upper limb weakness (34.37%), dysarthria (27.78%), and sensory loss (22.2%) (Figure S1).

### 3.3 Discharge outcomes

The medium length of stay was 12 days. The change in NIHSS from baseline to discharge was shown in Figure S2. Improvement, worsening, and no change in NIHSS occurred in 75.71% (399/527), 5.50% (29/527), and 18.79% (99/527) patients, respectively, in the IV t‐PA group and 51.7% (712/1377), 12.9% (178/1377), 35.37% (487/1377) patients, respectively, in the non‐IV t‐PA group before PSM. These factors were balanced after PSM. There was a significant difference in improvement in NIHSS at discharge by treatment status. Table 2 elucidated the association of IV t‐PA with discharge outcomes and three multivariable models were analyzed. After adjustment for age, sex, ethnicity, and baseline NIHSS, IV t‐PA was associated with improvement in NIHSS at discharge (adjusted odds ratio [aOR], 2.29; 95% confidence interval [CI], 1.78–2.95; *p* < 0.001). After adjustment plus medical insurance, onset to door time, arrival modality, current smoking, hypertension, diabetes, hyperlipidemia, prior stroke/TIA, AF, CHD, heart failure, carotid stenosis, PVD, anticoagulant, antiplatelet, antihypertensive, lipid‐lowering agents, glucose‐lowering agents, stroke unit, SBP at admission, and TOAST subtype, we found IV t‐PA was still associated with improvement in NIHSS at discharge (aOR, 2.26; 95% CI, 1.73–2.96; *p* < 0.001). In the aforementioned multivariable model plus imaging marker of sEICAS, a similar association of IV t‐PA with improvement in NIHSS was observed. All covariates were well‐balanced after the PSM (Table S1). In a propensity score analysis, the association of IV t‐PA with improvement in NIHSS was also identified (OR, 1.87; 95% CI, 1.40–2.51; *p* < 0.001).

**TABLE 2:** Association of alteplase with discharge outcomes in all subjects.

Model	Improvement in NIHSS *n* = 1111 (58.35%)		mRS score 0–1 *n* = 1546 (81.15%)	
	OR (95% CI)	*p* Value	OR (95% CI)	*p* Value
Univariate	2.91 (2.32–3.65)	<0.001	1.14 (0.87–1.48)	0.34
Model 1	2.29 (1.78–2.95)	<0.001	1.49 (1.13–1.96)	0.004
Model 2	2.26 (1.73–2.96)	<0.001	1.43 (1.06–1.92)	0.02
Model 3	2.23 (1.67–2.97)	<0.001	1.43 (1.04–1.96)	0.03
Propensity score matching	1.87 (1.40–2.51)	<0.001	1.46 (1.04–2.07)	0.03

*Note*: Model 1: adjusted for age, sex, ethnicity, and baseline NIHSS. Model 2: adjusted for all age, sex, ethnicity, baseline NIHSS, medical insurance, onset to door time, arrival modality, current smoking, hypertension, diabetes mellitus, hyperlipidemia, prior CHD, AF, heart failure, prior stroke/TIA, carotid stenosis, PVD, SBP at admission, stroke unit, antiplatelet, anticoagulant, antihypertensive, lipid‐lowering agents, glucose‐lowering agents, and TOAST subtype. Model 3: model 2 plus sEICAS ≥50%.

Abbreviations: AF, Atrial fibrillation; CHD, coronary heart disease; mRS, modified Rankin Scale; NIHSS, National Institutes of Health Stroke Scale; PVD, peripheral vascular disease; SBP, systolic blood pressure; sEICAS, symptomatic extra‐intracranial atherosclerotic stenosis; TIA, transient ischemic attack; TOAST, Trial of ORG 10172 in Acute Stroke Treatment.

At discharge, 81.15% (1546/1905) of participants had a favorable functional outcome (defined as mRS score ≤ 1). After adjustment for age, sex, ethnicity, and baseline NIHSS, IV t‐PA was associated with favorable functional outcomes at discharge (aOR, 1.49; 95% CI, 1.13–1.96; *p* = 0.004). In following different multivariable models and propensity score analysis, a similar association of IV t‐PA with the favorable functional outcome at discharge was observed (Table 2).

### 3.4 3‐, 6‐month, and 1‐year functional outcomes

Follow‐up was available for 1885 (98.95%) at 3 months, 1886 (99.0%) at 6 months, and 1871 (98.2%) at 1 year; a total of 73 (3.8%) were lost to follow‐up without 3‐ or 6‐month or 1‐year outcome (Table S2). Overall, 12.8% (242/1885) had a disabled outcome (defined as mRS score ≥ 2) at 3 months, 13.9% (262/1886) at 6 months, and 13.9% (260/1871) at 1 year. The unadjusted outcomes at 3, 6 months, and 1 year between the two groups were similar.

The associations of IV t‐PA with 3‐, 6‐month, and 1‐year functional outcomes were described in Table 3. There was no association between IV t‐PA and functional outcomes compared with non‐IV t‐PA in univariate analysis. After adjusting for age, sex, ethnicity, and baseline NIHSS, IV t‐PA was associated with 3‐, 6‐month, and 1‐year mRS scores of 0–1 (aOR, 1.51; 95% CI, 1.09–2.10; *p* = 0.01; aOR, 1.64; 95% CI, 1.19–2.27; *p* = 0.003; aOR, 1.52; 95% CI, 1.10–2.10; *p* = 0.01, respectively). After adjusting for age, sex, ethnicity, and baseline NIHSS plus medical insurance, onset‐to‐door time, arrival modality, hypertension, diabetes, hyperlipidemia, current smoking, prior stroke/TIA, AF, CHD, heart failure, carotid stenosis, PVD, anticoagulation, antiplatelet, antihypertensive, lipid‐lowering agents, glucose‐lowering agents, stroke unit, SBP at admission, and TOAST subtype, we found IV t‐PA was with a robust independent predictor for 3‐, 6‐month, and 1‐year mRS score of 0–1 (aOR, 1.46; 95% CI, 1.03–2.06; *p* = 0.03; aOR, 1.65; 95% CI, 1.17–2.33; *p* = 0.004; aOR, 1.51; 95% CI, 1.07–2.13; *p* = 0.02, respectively). In the aforementioned multivariable model plus imaging marker of sEICAS, a similar association of IV t‐PA with functional outcomes was observed. In a propensity score analysis, the associations of IV t‐PA with 3‐, 6‐month, and 1‐year mRS scores of 0–1 were also noted (OR, 1.54; 95% CI, 1.02–2.34; *p* = 0.04; OR, 1.91; 95% CI, 1.27–2.88; *p* = 0.002; OR, 1.61; 95% CI, 1.06–2.47; *p* = 0.03, respectively).

**TABLE 3:** Association of alteplase with 3‐, 6‐month, and 1‐year mRS 0–1 in all subjects.

Model	3‐month *n* = 1643 (87.16%)		6‐month *n* = 1624 (86.11%)		1‐year *n* = 1611 (86.10%)	
	OR (95% CI)	*p* Value	OR (95% CI)	*p* Value	OR (95% CI)	*p* Value
Univariate	1.21 (0.88–1.65)	0.24	1.36 (1.00–1.85)	0.05	1.32 (0.97–1.79)	0.08
Model 1	1.51 (1.09–2.10)	0.01	1.64 (1.19–2.27)	0.003	1.52 (1.10–2.10)	0.01
Model 2	1.46 (1.03–2.06)	0.03	1.65 (1.17–2.33)	0.004	1.51 (1.07–2.13)	0.02
Model 3	1.60 (1.10–2.33)	0.01	1.82 (1.25–2.65)	0.002	1.61 (1.06–2.47)	0.03
Propensity score matching	1.54 (1.02–2.34)	0.04	1.91 (1.27–2.88)	0.002	1.61 (1.06–2.47)	0.03

*Note*: Model 1: adjusted for age, sex, ethnicity, and baseline NIHSS. Model 2: adjusted for all age, sex, ethnicity, baseline NIHSS, medical insurance, onset to door time, arrival modality, current smoking, hypertension, diabetes mellitus, hyperlipidemia, prior CHD, AF, heart failure, prior stroke/TIA, carotid stenosis, PVD, SBP at admission, stroke unit, antiplatelet, anticoagulant, antihypertensive, lipid‐lowering agents, glucose‐lowering agents, and TOAST subtype. Model 3: model 2 plus sEICAS ≥50%.

Abbreviations: AF, Atrial fibrillation; CHD, coronary heart disease; mRS, modified Rankin Scale; NIHSS, National Institutes of Health Stroke Scale; PVD, peripheral vascular disease; SBP, systolic blood pressure; sEICAS, symptomatic extra‐intracranial atherosclerotic stenosis; TIA, transient ischemic attack; TOAST, Trial of ORG 10172 in Acute Stroke Treatment.

### 3.5 Hemorrhagic events

Eleven (2.1%) patients developed ICH in IV t‐PA group and 12 (0.9%) in non‐IV t‐PA group, of which 3 (0.6%) patients developed sICH in IV t‐PA group versus 2 (0.1%) in non‐IV t‐PA group. No fatal event was observed due to sICH. There was no significant difference in sICH rates by treatment status (Table 4).

**TABLE 4:** Safety events of patients based on intravenous alteplase.

Outcome	IV t‐PA *n* (%)	Non‐IV t‐PA *n* (%)	*p* Value
ICH within 36 h	11 (2.1)	12 (0.9)	0.03
sICH within 36 h	3 (0.6)	2 (0.1)	0.11
All‐cause mortality			
At discharge	0 (0)	1 (0.1)	0.54
At 3‐month	0 (0)	9 (0.7)	0.06
At 6‐month	4 (0.8)	14 (1.0)	0.60
At 1‐year	8 (1.5)	25 (1.8)	0.66

Abbreviations: ICH, intracerebral hemorrhage; IV t‐PA, intravenous tissue‐type plasminogen activator; sICH, symptomatic intracerebral hemorrhage.

### 3.6 All‐cause mortality

The rates of all‐cause mortality were no significant difference between IV t‐PA group and non‐IV t‐PA group at short‐, medium, and long‐term follow‐up periods (0 versus 1 [0.1%] at discharge; 0 versus 9 [0.7%] at 3‐month; 4 [0.8%] versus 14 [1.0%] at 6‐month; 8 [1.5%] versus 25 [1.8%] at 1‐year, respectively; Table 4).

### 3.7 Subgroup analysis

Subgroup analysis was shown in Figure S3. In subgroup analysis restricted to NIHSS score of 0–2, after adjusting for age, sex, ethnicity, and baseline NIHSS, the discrepant associations between IV t‐PA and mRS score of 0–1 at discharge (aOR, 1.52; 95% CI, 0.94–2.47; *p* = 0.09), 3 months (aOR, 1.67; 95% CI, 0.90–3.09; *p* = 0.11), 6 months (aOR, 1.76; 95% CI, 0.97–3.20; *p* = 0.06), and 1 year (aOR, 1.83; 95% CI, 1.03–3.27; *p* = 0.04) were observed. When restricted to an NIHSS score of 3–5, IV t‐PA was associated with favorable functional outcomes at discharge (aOR, 1.49; 95% CI, 1.06–2.08; *p* = 0.02) and 6 months (aOR, 1.58; 95% CI, 1.07–2.33; *p* = 0.02), while at 3 months and 1 year follow‐up time points, the association of IV t‐PA on favorable functional outcome was not observed. There was no interaction between baseline NIHSS and alteplase treatment with a better functional outcome (*p* = 0.90). When restricted to sEICAS ≥50%, after adjusting for age, sex, ethnicity, and baseline NIHSS, IV t‐PA was not associated with discharge, 3‐, 6‐month, and 1‐year favorable functional outcome. However, when restricted to a final diagnosis of ischemic stroke, the associations between IV t‐PA and favorable functional outcome at discharge (aOR, 1.53; 95% CI, 1.16–2.02; *p* = 0.002), 3 months (aOR, 1.52; 95% CI, 1.10–2.11; *p* = 0.01), 6 months (aOR, 1.66; 95% CI, 1.20–2.30; *p* = 0.002), and 1 year (aOR, 1.53; 95% CI, 1.11–2.12; *p* = 0.01) were identified.

## 4 DISCUSSION

Our study found that discharge, 3‐, 6‐month, and 1‐year favorable functional outcomes occurred in more than 80% of minor stroke patients with NIHSS score of 0–5 within 4.5 h from the onset, accompanied by a low rate of sICH. Also, we identified IV t‐PA was associated with favorable functional outcomes in the overall cohort.

Previous studies have indicated that minor strokes might be related to greater probabilities of impairment. The Get With The Guidelines Stroke (GWTG‐S) registry which was an American quality improvement program including 5910 patients with a baseline NIHSS score of 0–5 treated with IV t‐PA reported over 30% could not ambulate independently at discharge.^16^ Another retrospective analysis from the GWTG‐S registry described 25% of patients with an NIHSS score of 0–5 not treated with thrombolysis could not be released to ambulate independently or straight to home.^17^ The Mild and Rapidly Improving Stroke Study (MaRISS) which was a prospective observational study including 1765 minor stroke patients with a baseline NIHSS score of 0–5 reported 21% of patients were unable to ambulate independently at discharge and 37% had a disabled outcome at 3 months.^4^ Nevertheless, we found the rate of disabled outcome was less than 20% in the short‐, medium‐ or long‐term follow‐up, which was lower than that in the abovementioned observational studies. It was in accordance with our previous study from the Chinese Stroke Center Alliance (CSCA), which was similar to the GWTG‐S registry and included 6752 minor stroke patients (NIHSS ≤ 5) treated with IV t‐PA reported only 10.1% could not ambulate independently at discharge.^8^ The potential causes of the low disabled rate might encompass the longer length of stay, early intervention of rehabilitation, race or ethnicity, etc.^8^, ^18^

The proportion of thrombolysis in our study was 28%, which was similar to 25% alteplase administration in minor stroke reported in large population‐based studies.^19^ It meant more than 70% of patients with minor stroke did not receive thrombolysis, even if they presented within the 4.5‐h window and had no contraindications for treatment. Potential reasons for this low thrombolysis rate might be related to low NIHSS score, being perceived as rapidly improving, and being afraid of intracerebral hemorrhage risk.

We also observed that IV t‐PA was associated with short‐, medium, and long‐term favorable functional outcomes in the overall cohort compared with non‐IV t‐PA. Our findings of the association of IV t‐PA and short‐ and medium‐functional outcomes were in conformity with some observational studies.^3^, ^11^, ^20^, ^21^ A Korean retrospective analysis based on a multicenter registry database including 1384 minor stroke patients (of 194 in IV t‐PA group) showed IV t‐PA was associated with higher odds of a favorable outcome at 3 months compared with non‐IV tPA.^11^ A study from the Austrian Stroke Unit Registry including 890 minor stroke patients (of 445 in t‐PA group) suggested IV t‐PA treatment might be beneficial for patients with mild neurological deficits and the numbers need to treat 8–14.^21^ Another retrospective study using the United States inpatient database including 103,765 minor stroke patients (of 10,300 in IV t‐PA alone) found intravenous thrombolysis was independently associated with discharge directly to home without assistance.^3^ A meta‐analysis including seven studies with 1591 patients, in which minor stroke defined as NIHSS ≤ 6, indicated IV t‐PA was associated with better functional outcomes at 3 or 6 months.^20^ The post hoc analysis of the Efficacy and Safety of MRI‐based Thrombolysis in Wake‐up Stroke (WAKE‐UP) trial^22^ showed IV t‐PA brought an absolute increase of 12% of 90‐day favorable functional outcome compared with placebo in patients with lacunar infarcts and the median NIHSS score of five points on admission.^23^ However, the only phase three randomized clinical trial of minor stroke PRISMS did not find IV t‐PA increased the probability of a favorable functional outcome at 3 months compared with aspirin.^7^ As noted, this trial only enrolled 313 patients due to termination early and was underpowered to draw a convincing conclusion. Moreover, this trial was designed for minor non‐disabling ischemic stroke, with predominately very mild patients (62.2% of patients had baseline NIHSS score of 0–2) in the alteplase group. In comparison, our study included a wide spectrum of mild stroke patients, with 44.6% of patients having a baseline NIHSS score of 0–2. Another post hoc analysis of multiple databases with different study purposes showed no significant difference was detected between IV t‐PA and favorable functional outcome at 3 months. In this study, minor stroke was defined by an NIHSS score of 0–3, which was different from our study.^24^ In addition to demographic profiles, the underlying reasons for this discrepancy in results might include thrombus characteristics, early recanalization, hemispheric cerebral blood flow, etc.^25^, ^26^ Besides, so far, the association between IV t‐PA and long‐term functional outcome in minor stroke patients was seldom reported. Our study provided the rate of 1‐year favorable functional outcome (86.1%) in minor stroke patients. Furthermore, the association of IV t‐PA with functional outcome was still observed at 1 year and IV t‐PA was associated with 1‐year favorable functional outcome, compared with non‐IV t‐PA.

In the subgroup analyses, we found IV t‐PA was statistically associated with 90‐day mRS 0–1 in the baseline NIHSS of 3–5 group, while the association was borderline significant in the baseline NIHSS of 0–2 subgroup. Similarly, the MaRISS study found alteplase was associated with a favorable 3‐month outcome of Stroke Impact Scale‐16 in the prespecified subgroup of baseline NIHSS score of 3–5.^4^ We noticed the percentage with an NIHSS score of 0–2 in our study was numerically higher than that in the MaRISS alteplase‐treated group (36.2%). However, the syndromic severity of symptoms was different between the two studies. The patients in our study were more likely to have lower and upper limb weakness and less likely to have dysarthria and sensory loss as compared with patients in MaRISS. Another observational study from the Austrian multicenter stroke registry showed patients with NIHSS score of 2–5, IV t‐PA was associated with a higher rate of favorable functional outcomes at 3 months.^2^ In addition, our study showed patients with NIHSS score of 0–2 seemed not to be sensitive to IV t‐PA treatment compared with patients with NIHSS score of 3–5. Although we initially did not categorize the patients into disabling and non‐disabling based on neurologic deficits and patients with NIHSS score of 0–5 constituted a heterogeneous group, patients with NIHSS score of 0–2 might indicate very mild neurologic deficits, including more non‐disabling strokes, in which treatment with alteplase versus aspirin did not improve functional outcome at 3 months from PRISMS trial. A recent retrospective study showed mild neurologic deficits might mean less penumbra and lower hemispheric cerebral blood flow, and even given reperfusion, the benefit was limited,^25^ which needed further validation. Also, in the NIHSS score of 0–2 subgroup, we observed the effect size of IV t‐PA on functional outcome was enlarged from 3‐, 6‐month to 1‐year follow‐up, which was not obvious in the NIHSS score of 3–5 subgroup. The recent subsequent study from the MaRISS population exhibited 17% of patients improved on the mRS between 1‐ and 3‐month follow‐up term poststroke which meant a minor stroke might induce a greater possibility of longer‐term functional improvement.^27^ However, this has to be validated in future studies. Our study also found a favorable trend but no significant difference of IV t‐PA on functional outcome in the restricted sample of sEICAS ≥ 50%, partly because of the limited sample size (*n* = 124).

In our study, although the overall rate of ICH within 36 hours was higher in patients with IV t‐PA than non‐IV t‐PA group, the rate of sICH was comparable and no significant difference was observed between the two groups. The risk of sICH in our study was low and in line with the previous studies.^4^, ^16^, ^20^ Furthermore, IV t‐PA did not increase the rate of all‐cause mortality.

### 4.1 Limitations

Our study also has limitations. First, it has potential bias out of a cohort study, which was based on hospitals' voluntary and convenience in nature. However, the CNSR‐III study covered 201 hospitals in 22 provinces and four municipalities and was representative of Chinese clinical practice. Second, we did not differentiate disabling from non‐disabling minor strokes attributed to lacking these variables in the CNSR‐III database. Third, the limited sample in the subgroups underpowered the association between IV t‐PA and functional outcomes at different follow‐up terms. Fourth, due to heterogeneity and inadequate drug compliance in clinical practice, our study is only an exploratory analysis based on the registry cohort and the results need to be verified in randomized controlled trials. The ongoing ARAMIS (Antiplatelet vs R‐tPA for Acute Mild Ischemic Stroke) (NCT03661411),^28^ TEMPO‐2 (A Randomized Controlled Trial of TNK‐tPA Versus Standard of Care for Minor Ischemic Stroke With Proven Occlusion) (NCT02398656), and PUMICE (ProUrokinase in Mild IsChemic strokE) (NCT05507645) trial will hopefully provide more robust evidence of thrombolysis in minor stroke.

## 5 CONCLUSIONS

Our findings suggest minor stroke with NIHSS score of 0–5 treated with IV t‐PA, as compared to non‐IV t‐PA, was associated with short‐, medium‐, and long‐term favorable functional outcomes with infrequent sICH. Further large randomized controlled trials are warranted.

## AUTHOR CONTRIBUTIONS

Chunmiao Duan and Yunyun Xiong contributed equally. Manuscript draft and editorial design—Chunmiao Duan and Yunyun Xiong. Statistical analysis—Hong‐Qiu Gu and Kai‐Xuan Yang. Critical revision of the manuscript—Shang Wang and Manjun Hao. Project supervision—Xingquan Zhao and Mia Meng. Editorial design and funding acquisition—Yongjun Wang.

## FUNDING INFORMATION

This work was supported by grants from the Capital's Funds for Health Improvement and Research (2020‐1‐2041), Chinese Academy of Medical Sciences Innovation Fund for Medical Sciences (2019‐I2M‐5‐029), National Natural Science Foundation of China (81,870,905, U20A20358, 82,171,272), Beijing Municipal Science & Technology Commission (Z211100003521019), Beijing Hospitals Authority (PX2022019).

## CONFLICT OF INTEREST STATEMENT

The authors declare no conflict of interest.

## Supporting information


Appendix S1

## Data Availability

The data supporting the study's conclusions are accessible from the respective authors upon reasonable request.
