# Supplementary material for: Outcomes in minor stroke patients treated with intravenous thrombolysis
Source: CNS Neurosci Ther. 2023 Mar 21;29(8):2308–17. doi: 10.1111/cns.14164 (PMC10352890; doi:10.1111/cns.14164)
Supplement: Supplementary file 1 — Appendix S1 [file CNS-29-2308-s001.docx]

**Supplementary material**

**Supplementary Table 1.** Baseline Characteristics of Patients after Propensity Score Matching

**Supplementary Table 2.** Follow-up Functional Outcomes at 3, 6 Months and 1 Year in Whole Cohort

**Supplementary Figure 1.** The Distribution of Subitems on The Baseline NIHSS

**Supplementary Figure 2.** Change in National Institutes of Health Stroke Scale from Baseline to Discharge by Intravenous Alteplase Treatment before and after PSM

**Supplementary Figure 3.** Association of Alteplase with Discharge, 3-, 6-Month and 1-Year Functional Outcomes in Subgroups

**Supplementary Table 1. Baseline Characteristics of Patients after Propensity Score Matching**

| Variables | Total | Non-IV t-PA | IV t-PA | *P* value |
| --- | --- | --- | --- | --- |
|  | (n=860) | (n=430) | (n=430) |  |
| Age, y | 63.0 (55.0-70.0) | 63.0 (55.0-70.0) | 62.0 (55.0-69.0) | 0.82 |
| Female | 261 (30.3) | 127 (29.5) | 134 (31.2) | 0.60 |
| Ethnicity |  |  |  | 0.82 |
| Han | 841 (97.8) | 421 (97.9) | 420 (97.7) |  |
| Non-Han | 19 (2.2) | 9 (2.1) | 10 (2.3) |  |
| Medical insurance |  |  |  |  |
| Urban worker/public health medical insurance | 436 (50.7) | 218 (50.7) | 218 (50.7) | 1.00 |
| Urban resident basic medical insurance | 156 (18.1) | 80 (18.6) | 76 (17.7) | 0.72 |
| Rural cooperation medical insurance | 210 (24.4) | 102 (23.7) | 108 (25.1) | 0.63 |
| Commercial insurance | 4 (0.5) | 3 (0.7) | 1 (0.2) | 0.32 |
| Self-payment | 63 (7.3) | 31 (7.2) | 32 (7.4) | 0.90 |
| Time measure |  |  |  | 0.24 |
| Onset to door, hours | 2.0 (1.0-2.7) | 2.0 (1.0-2.8) | 1.9 (1.0-2.6) |  |
| ≤3h | 720 (83.7) | 357 (83.0) | 363 (84.4) |  |
| 3-4.5h | 140 (16.3) | 73 (17.0) | 67 (15.6) |  |
| Arrival modality |  |  |  | 0.84 |
| Arrival by EMS | 174 (20.2) | 84 (19.5) | 90 (20.9) |  |
| Arrival self | 514 (59.8) | 261 (60.7) | 253 (58.8) |  |
| Arrival others | 172 (20.0) | 85 (19.8) | 87 (20.2) |  |
| Medical history |  |  |  |  |
| Current smoking | 280 (32.6) | 140 (32.6) | 140 (32.6) | 1.00 |
| Hypertension | 561 (65.2) | 290 (67.4) | 271 (63.0) | 0.17 |
| Diabetes mellitus | 180 (20.9) | 91 (21.2) | 89 (20.7) | 0.87 |
| Dyslipidemia | 67 (7.8) | 35 (8.1) | 32 (7.4) | 0.70 |
| Prior CHD | 79 (9.2) | 42 (9.8) | 37 (8.6) | 0.56 |
| AF | 72 (8.4) | 38 (8.8) | 34 (7.9) | 0.62 |
| Heart failure | 3 (0.3) | 2 (0.5) | 1 (0.2) | 0.56 |
| Prior Stroke/TIA | 156 (18.1) | 84 (19.5) | 72 (16.7) | 0.29 |
| Carotid stenosis | 6 (0.7) | 2 (0.5) | 4 (0.9) | 0.41 |
| PVD | 2 (0.2) | 1 (0.2) | 1 (0.2) | 1.00 |
| Fasting glucose at admission, mmol/L | 5.6 (4.9-6.8) | 5.7 (5.0-6.8) | 5.5 (4.9-6.8) | 0.12 |
| SBP at admission, mmHg | 151.0 (139.5-166.0) | 151.5 (138.5-165.5) | 151.0 (140.0-166.0) | 0.99 |
| DBP at admission, mmHg | 87.0 (80.0-97.5) | 87.0 (80.0-98.0) | 87.5 (79.5-95.5) | 0.74 |
| Baseline NIHSS | 3.0 (1.0-4.0) | 3.0 (1.0-4.0) | 3.0 (1.0-4.0) | 0.91 |
| 0 | 83 (9.7) | 49 (11.4) | 34 (7.9) |  |
| 1 | 156 (18.1) | 76 (17.7) | 80 (18.6) |  |
| 2 | 167 (19.4) | 73 (17.0) | 94 (21.9) |  |
| 3 | 159 (18.5) | 82 (19.1) | 77 (17.9) |  |
| 4 | 179 (20.8) | 91 (21.2) | 88 (20.5) |  |
| 5 | 116 (13.5) | 59 (13.7) | 57 (13.3) |  |
| Care in stroke unit | 345 (40.1) | 173 (40.2) | 172 (40.0) | 0.94 |
| TOAST subtype |  |  |  | 0.94 |
| LAA | 193 (22.4) | 93 (21.6) | 100 (23.3) |  |
| Cardioembolic | 80 (9.3) | 43 (10.0) | 37 (8.6) |  |
| SAO | 197 (22.9) | 98 (22.8) | 99 (23.0) |  |
| Other determined cause | 11 (1.3) | 6 (1.4) | 5 (1.2) |  |
| Undetermined cause | 379 (44.1) | 190 (44.2) | 189 (44.0) |  |
| Imaging marker |  |  |  |  |
| sEICAS ≥ 50% | 221 (25.7) | 107 (24.9) | 114 (26.5) | 0.58 |
| Medication history |  |  |  |  |
| Antiplatelet | 123 (14.3) | 69 (16.0) | 54 (12.6) | 0.14 |
| Anticoagulant | 5 (0.6) | 3 (0.7) | 2 (0.5) | 0.65 |
| Antihypertensive | 410 (47.7) | 215 (50.0) | 195 (45.3) | 0.17 |
| Lipid lowering agents | 81 (9.4) | 43 (10.0) | 38 (8.8) | 0.56 |
| Glucose lowering agents | 143 (16.6) | 74 (17.2) | 69 (16.0) | 0.65 |

EMS indicates emergency medical services; CHD, coronary heart disease; AF, atrial fibrillation; TIA, transient ischemic attack; PVD, peripheral vascular disease; SBP, systolic blood pressure; DBP, diastolic blood pressure; NIHSS, National Institutes of Health Stroke Scale; TOAST, Trial of ORG 10172 in Acute Stroke Treatment; LAA, large artery atherosclerosis; SAO, small artery occlusion; sEICAS, symptomatic extra-intracranial atherosclerotic stenosis; IV t-PA, intravenous tissue-type plasminogen activator.

**Supplementary Table 2. Follow-up Functional Outcomes at 3, 6 Months and 1 Year in Whole Cohort**

| outcome | 3-month  (n=1,885) | 6-month  (n=1,886) | 1-year  (n=1,871) |
| --- | --- | --- | --- |
| mRS score 0, % | 44.14 | 49.26 | 51.26 |
| mRS score 1, % | 43.02 | 36.85 | 34.85 |
| mRS score 2, % | 7.53 | 7.69 | 6.95 |
| mRS score 3, % | 3.13 | 2.60 | 3.10 |
| mRS score 4, % | 1.22 | 1.11 | 1.60 |
| mRS score 5, % | 0.37 | 0.69 | 0.43 |
| mRS score 6, % | 0.58 | 1.80 | 1.82 |

mRS indicates modified Rankin Scale.

**Supplementary Figure 1. The Distribution of Subitems on The Baseline NIHSS**


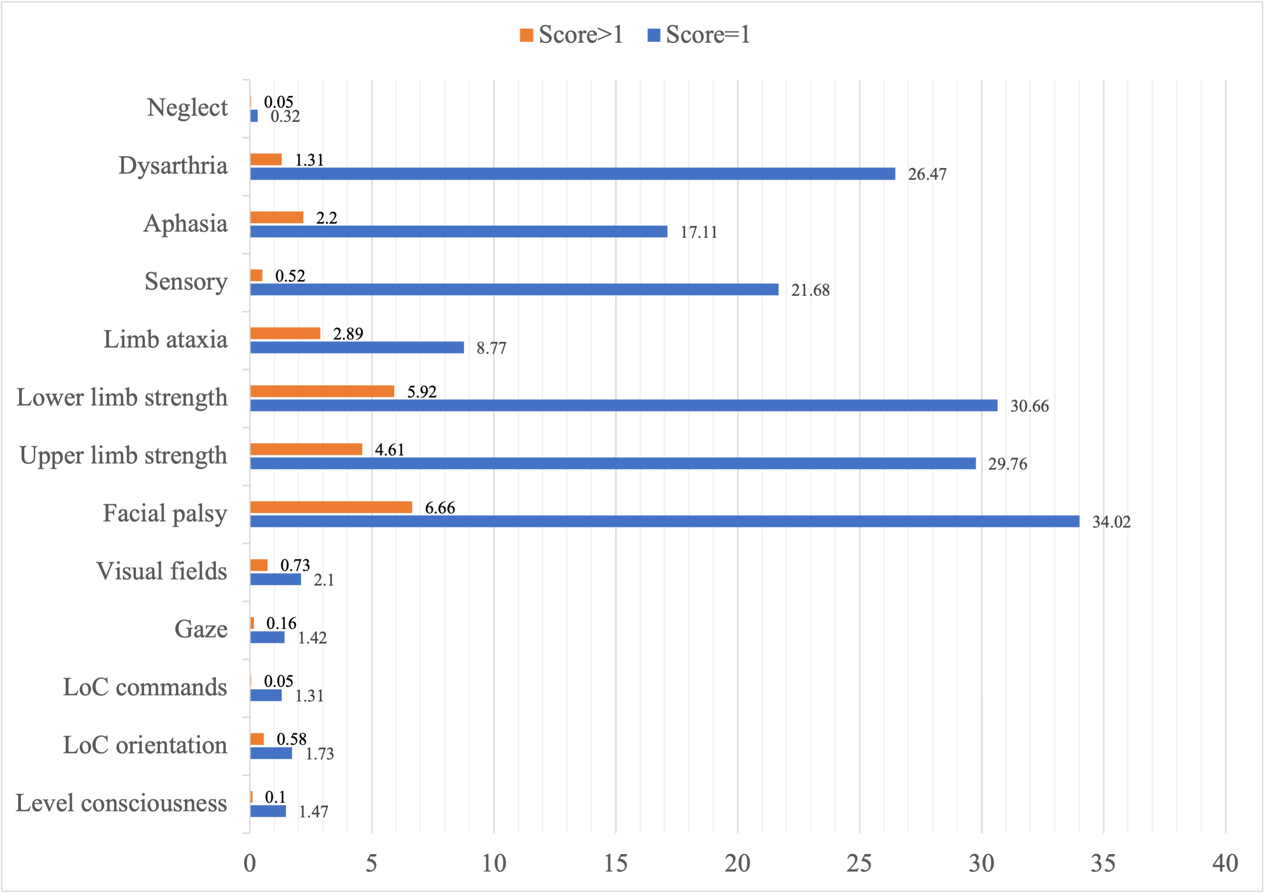


**Supplementary Figure 2. Change in National Institutes of Health Stroke Scale from Baseline to Discharge by Intravenous Alteplase Treatment before and after PSM**


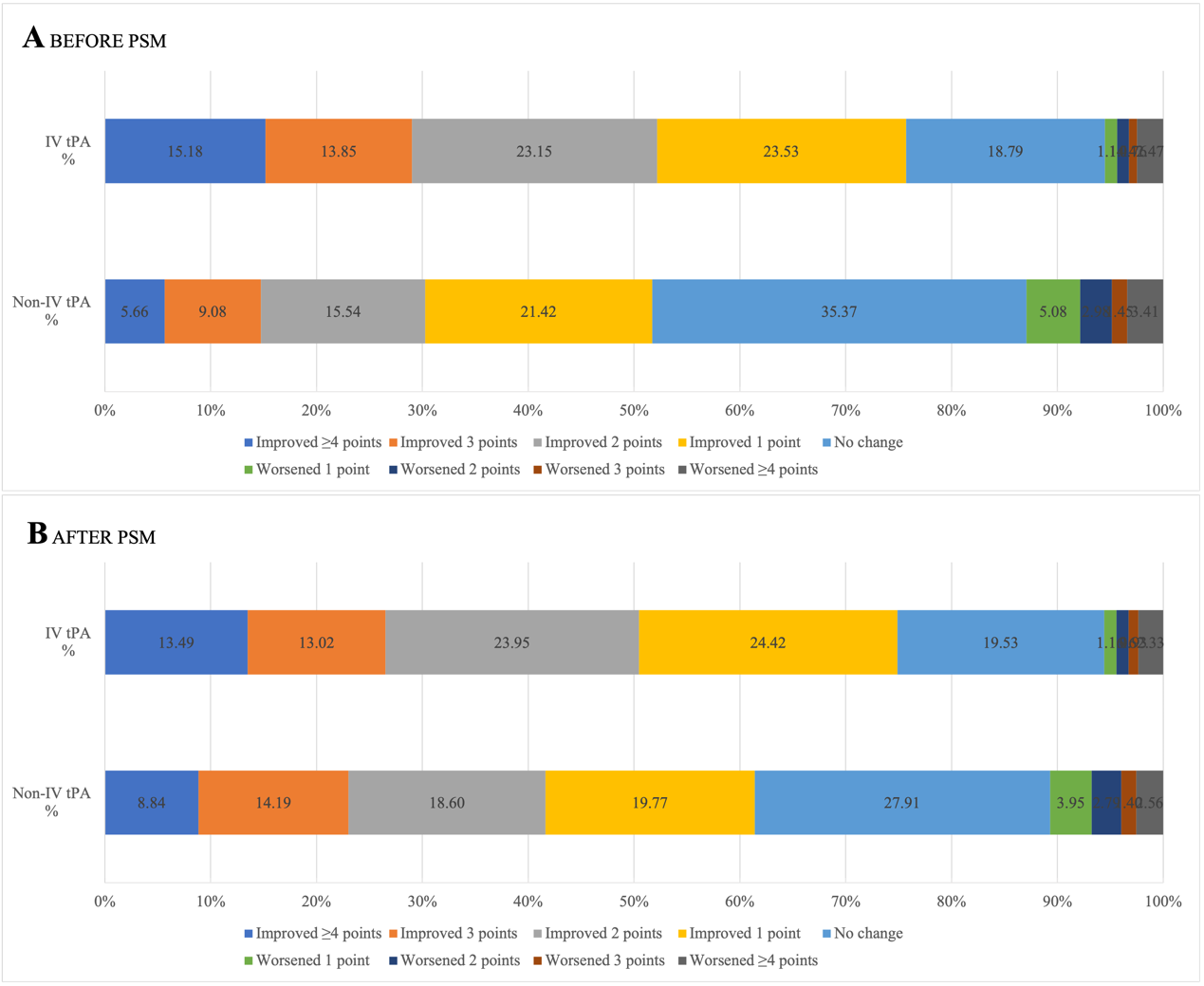


**Supplementary Figure 3. Association of Alteplase with Discharge, 3-, 6-Month and 1-Year Functional Outcomes in Subgroups**


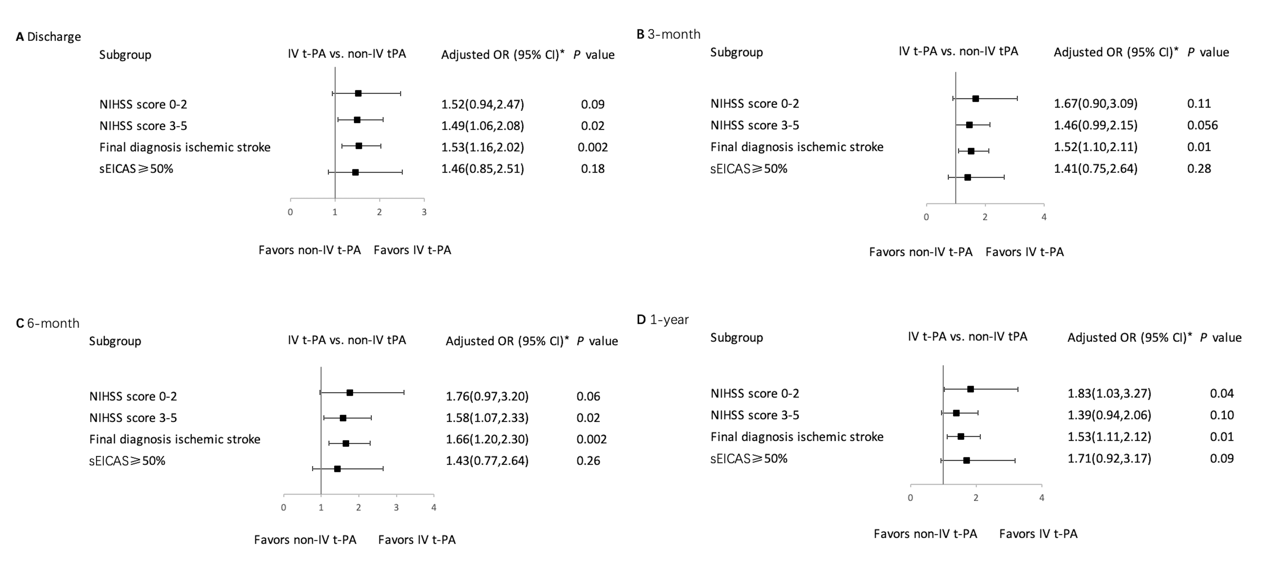


NIHSS indicates National Institutes of Health Stroke Scale; CHD, coronary heart disease; AF, Atrial fibrillation; TIA, transient ischemic attack; PVD, peripheral vascular disease; SBP, systolic blood pressure; TOAST, Trial of ORG 10172 in Acute Stroke Treatment; sEICAS, symptomatic extra-intracranial atherosclerotic stenosis; IV t-PA, intravenous tissue-type plasminogen activator.

*Adjusted for age, sex, ethnicity, and baseline NIHSS. Similar results when adjusted for all age, sex, ethnicity, baseline NIHSS, medical insurance, onset to door time, arrival modality, current smoking, hypertension, diabetes mellitus, hyperlipidemia, prior CHD, AF, heart failure, prior stroke/TIA, carotid stenosis, PVD, SBP at admission, stroke unit, antiplatelet, anticoagulant, antihypertensive, lipid lowering agents, glucose lowering agents, and TOAST subtype with or without sEICAS ≥ 50%.
